# Association Between CD4/CD8 Ratio Recovery and Chronic Kidney Disease Among Human Immunodeficiency Virus-Infected Patients Receiving Antiretroviral Therapy: A 17-Year Observational Cohort Study

**DOI:** 10.3389/fmicb.2022.827689

**Published:** 2022-02-10

**Authors:** Fengxiang Qin, Qing Lv, Wen Hong, Di Wei, Kui Huang, Ke Lan, Rongfeng Chen, Jie Liu, Bingyu Liang, Huayue Liang, Hao Liang, Shanfang Qin, Li Ye, Junjun Jiang

**Affiliations:** ^1^Guangxi Key Laboratory of AIDS Prevention and Treatment, School of Public Health, Guangxi Medical University, Nanning, China; ^2^Guangxi Collaborative Innovation Center for Biomedicine, Life Sciences Institute, Guangxi Medical University, Nanning, China; ^3^Chest Hospital of Guangxi Zhuang Autonomous Region, Liuzhou, China

**Keywords:** HIV, AIDS, CD4/CD8, chronic kidney disease, antiretroviral therapy

## Abstract

**Background:**

CD4/CD8 ratio is considered as an emerging biomarker for human immunodeficiency virus (HIV)-related diseases. However, the relationship of CD4/CD8 ratio recovery and chronic kidney disease (CKD), and whether cumulative antiretroviral therapy (ART) is effective in the CD4/CD8 ratio recovery and in reducing CKD incidence among HIV patients remain unclear.

**Methods:**

A 17-year observational cohort study was conducted on all HIV-infected patients receiving ART in Guangxi, China. Kaplan–Meier analysis was used to investigate the cumulative CKD incidence. Cox regression and propensity score matching (PSM) were used to evaluate the association between CD4/CD8 ratio recovery and CKD incidence, and the effect of ART regimens on CD4/CD8 ratio recovery and CKD incidence.

**Results:**

A total of 59,268 eligible individuals contributing 285,143 person-years of follow-up, with an overall CKD incidence of 9.65%. After ART, patients who developed CKD showed higher mortality than those with normal kidney function (12.48 vs. 7.57%, *p* < 0.001). Patients whose CD4/CD8 ratio did not recover to 0.7 had a higher CKD incidence than the patients who recovered (aHR = 2.84, 95% CI 2.63–3.07), similar to the PSM analysis (aHR = 3.13, 95% CI 2.85–3.45). Compared with the PI-based and INSTI-based regimens, NNRTI-based regimen had a better CD4/CD8 ratio recovery rate (27.04, 16.16, and 29.66%, respectively) and a lower CKD incidence (17.43, 16.16, and 7.31%, respectively).

**Conclusion:**

This large-scale real-world setting provide new evidence that the CD4/CD8 ratio recovery is associated with lower CKD incidence in HIV-infected patients receiving ART. NNRTI-based is a better choice for CD4/CD8 ratio recovery and reducing the risk of CKD.

## Introduction

Antiretroviral therapy (ART) has significantly decreased the mortality of human immunodeficiency virus (HIV)/acquired immune deficiency syndrome (AIDS) patients. As patients live longer, chronic non-communicable diseases (NCDs) have become a prominent public health problem. Previous studies have shown that diabetes, hypertension, and chronic kidney disease (CKD) are important NCDs among HIV patients (Kooij et al., 2017; Mathabire Rucker et al., 2018; Sutton et al., 2019). In China, the CKD prevalence among HIV patients is approximately 16.1–16.8% (Cheung et al., 2007; Cao et al., 2013), which is slightly higher than that in Japan (15.4%) (Yanagisawa et al., 2011) and the United States (15.5%) (Wyatt et al., 2007). The kidney plays an important role in drug metabolism and excretion, and its function or deficiency thereof is more evident in HIV-infected patients receiving long-term ART. It is worth noting that persistent kidney damage leads to an increased risk of death (Cristelli et al., 2017; Ekrikpo et al., 2018). The Guangxi Zhuang Autonomous Region, located in western China, suffers a high burden of HIV infection, with more than 110,000 HIV patients receiving ART in 2020. However, no reports were available on the incidence of CKD in this population in Guangxi.

Low CD4 cell counts and high viral loads have been considered as influencing factors for CKD in HIV-infected patients (Jose et al., 2018; Brito et al., 2019). CD4 cell count is the most important index used to evaluate the immune function of HIV-infected patients. In contrast to a persistent decrease in CD4 cell count, CD8 cell count is persistently high in HIV-infected patients. With ART, the CD4 cell count in most patients gradually normalizes, but their CD8 cell count remains at a high level (Geltzeiler et al., 2020). This ultimately leads to the CD4/CD8 ratio remaining low and difficult to recover to normal levels. A cohort study conducted in Hong Kong, China indicated that only 33% of HIV patients achieved a CD4/CD8 ratio ≥0.8 even though the CD4 cell count reached 500 cells/μL or higher after ART (Lee et al., 2017). Similarly, another study in Italy indicated that only 29% of HIV patients treated with ART who achieved viral suppression reached a CD4/CD8 ratio ≥1.0 (Mussini et al., 2015). Some previous studies have shown that the CD4/CD8 ratio is independently associated with mortality and morbidity of immune dysfunction, and metabolic syndrome (Sigel et al., 2017; Trickey et al., 2017; Han et al., 2018; Castilho et al., 2019; Gojak et al., 2019). Therefore, the CD4/CD8 ratio has been considered as an emerging biomarker for HIV-related diseases in recent years (Serrano-Villar and Deeks, 2015). The recovery of the CD4/CD8 ratio in HIV-infected patients in Guangxi after ART has not been reported so far; in particular, the relationship between the CD4/CD8 ratio and CKD remains unclear.

Cumulative ART significantly decreases the estimated glomerular filtration rate (eGFR) of HIV patients and increases the risk of developing CKD (Yombi et al., 2014; Mocroft et al., 2016). Additionally, it has been reported that antiretrovirals have a certain influence on the recovery of the CD4/CD8 ratio in HIV-infected patients (Herrera et al., 2020; Serrano-Villar et al., 2020). We suspect that there may be associations among ART regimens, CD4/CD8 ratio recovery, and the development of CKD in HIV patients. In this study, we retrospectively collected data on HIV patients receiving ART in Guangxi from the Chinese National Free Antiretroviral Treatment Program (NFATP) database to evaluate the incidence of CKD and the recovery of the CD4/CD8 ratio among HIV patients receiving ART who initially had a normal eGFR and to investigate the association of the CD4/CD8 ratio recovery and CKD among these patients with different ART regimens.

## Materials and Methods

### Study Site and Participants

We retrospectively collected data from December 2003 through October 2020 for all HIV-infected adults reported to the NFATP at the initiation of ART in Guangxi, China. Patients who had a normal baseline eGFR (≥90 mL/min per 1.73 m^2^), available CD4/CD8 ratio measurements, treatment with ART, and triple treatment regimens [two nucleoside reverse transcriptase inhibitors (NRTIs) plus either a non-nucleoside reverse transcriptase inhibitor (NNRTI) (NNRTI-based), a protease inhibitor (PI) (PI-based), or an integrase strand transfer inhibitor (INSTI) (INSTI-based)] were included. Patients were excluded if they met the following criteria: (i) had no baseline CD4 cell count and/or CD8 cell count, (ii) did not have information to calculate baseline and follow-up eGFR, (iii) had a baseline eGFR <90 mL/min per 1.73 m^2^, (iv) were followed up for less than 6 months, (v) had a baseline CD4/CD8 ratio ≥1.0, or (vi) were treated with regimens other than NNRTI-based, PI-based and INSTI-based regimens. This study was approved by the Human Research Ethics Committee of Guangxi Medical University (Ethical Review No. 2019-SB-102).

### Definitions

Baseline was defined as various demographic data or clinical indicators from the most recent record prior to or following ART started. eGFRs were calculated by creatinine clearance as [(186 × serum creatinine)−(1.154 × age)−0.203] (for males) and [(186 × serum creatinine)−(1.154 × age) −(0.203 × 0.742]] (for females) (Simplified MRDR formula). CKD was defined as eGFR lower than 60 mL/min per 1.73 m^2^ (Mocroft et al., 2016) and defined as the outcome event. A CD4/CD8 ratio ≥0.7 at two consecutive visits after long-term ART was defined as the CD4/CD8 ratio recovery in our study.

### Statistics

Categorical variables were described as frequency while quantitative variables were expressed as the median ± interquartile range (IQR). A Kaplan–Meier graph was used to show the accumulative CKD incidence during the ART period, and statistical testing of differences was performed using the log-rank test. Multivariable Cox regression analysis was used to evaluate the association of CD4/CD8 ratio recovery and CKD incidence or ART regimen and CD4/CD8 ratio recovery or ART regimen combined with CD4/CD8 ratio recovery and CKD incidence. In addition, 1:1 propensity score matching (PSM) was used to match the characteristic variables between the CD4/CD8 ratio recovered and unrecovered groups, and 1:1:1 PSM was used to match the characteristic variables among the NNRTI-based, PI-based and INSTI-based groups. The forest plot was used to indicate the effect of the whole research population and the PSM population. Data were analyzed using the Statistical Package for the Social Sciences (SPSS) version 23.0 (SPSS Inc., Chicago, IL, United States), R studio (version 3.6.1; R studio, Boston, MA, United States) and GraphPad Prism version 8.2 (GraphPad Software, San Diego, CA, United States). A two-tailed statistical test with a *P-*value of 0.05 or less was considered statistically significant.

## Results

### Demographic Characteristics of Eligible Human Immunodeficiency Virus Patients at Baseline

**FIGURE 1:**
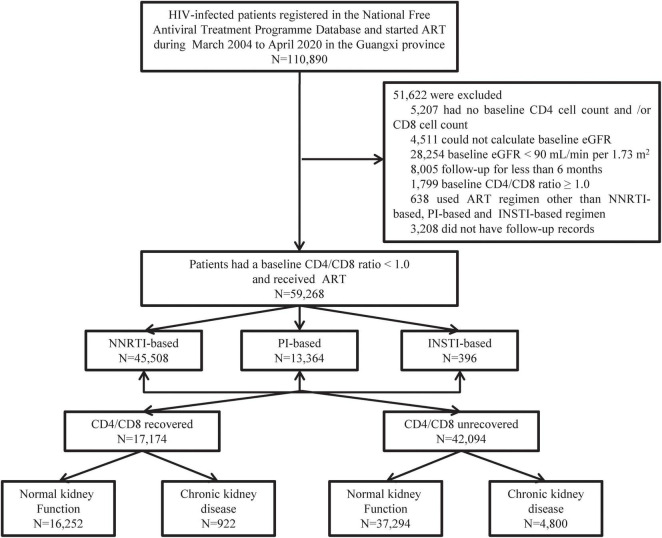
Patient enrollment flowchart.

A total of 110,890 HIV patients who initiated ART between December 2003 and October 2020 in Guangxi, China, were screened for inclusion in the present study. Of those patients, 51,622 were excluded, detail in Figure 1. Ultimately, 59,268 patients met the inclusion criteria and were included in this study. These eligible patients contributed 285,143 person-years of follow-up, with a median of 4 person-years (IQR 2–7). Table 1 shows that 64.63% patients were male, 63.49% were married or cohabiting, and most patients were diagnosed with HIV (63.71%) or started ART (65.79%) at 30–59 years of age. A total of 85.57% acquired HIV through heterosexual transmission, 45.65% had World Health Organization (WHO) HIV clinical stage I disease, 48.37% had a baseline body mass index (BMI) in the normal range (18.5–23.9 kg/m^2^). 53.30% patients had a CD4 cell count <200 cells/μL, 47.02% had a CD8 cell count ≤760 cells/μL. And 68.96% patients had a baseline CD4/CD8 ratio <0.30.

**TABLE 1:** Characteristics of HIV patients receiving ART [*n* (%)].

Variable	Study population (*n* = 59,268) (*n*, %)
**Sex**	
Male	38,304 (64.63)
Female	20,964 (35.37)
**Marital status**	
Single, divorced or widowed	21,482 (36.25)
Married or cohabitation	37,629 (63.49)
Unknown	157 (0.26)
Age at HIV diagnosis, year-old #	41.38 (31.57–54.34)
<30	12,235 (20.64)
30–59	37,758 (63.71)
≥60	9,275 (15.65)
Age at ART initiation, year-old #	42.12 (32.55–54.67)
<30	10,640 (17.95)
30–59	38,990 (65.79)
≥60	9,638 (16.26)
**HIV transmission route**	
Blood or plasma transfusion	3,820 (6.45)
Homosexual transmission	3,438 (5.80)
Heterosexual transmission	50,717 (85.57)
Other/unknown	1,293 (2.18)
**WHO HIV disease stage**	
I	27,055 (45.65)
II	9,206 (15.53)
III	10,264 (17.32)
IV	12,283 (20.72)
Unknown	460 (0.78)
Baseline body mass index, kg/m^2^ #	20.32 (18.66–22.39)
<18.5	10,613 (17.91)
18.5–23.9	28,668 (48.37)
24–27.9	4,783 (8.07)
≥28	900 (1.52)
Unknown	14,304 (24.13)
Baseline CD4 cell count, cells/μL #	183.82 (51.39–309.71)
<200	31,590 (53.3)
200–349	16,845 (28.42)
350–499	7,427 (12.53)
≥500	3,406 (5.75)
Baseline CD8 cell count, cells/μL #	795.37 (509.96–1166.44)
≤760	27,869 (47.02)
761–1138	15,745 (26.57)
≥1138	15,654 (26.41)
Baseline CD4/CD8 #	0.20 (0.08–0.34)
<0.30	40,874 (68.96)
0.3–0.69	16,694 (28.17)
0.70–0.99	1,700 (2.87)

*#Data are presented as medium [interquartile range (IQR)].*

### Kidney Function Was Related to Mortality in Human Immunodeficiency Virus Patients Receiving Antiretroviral Therapy

Of the 59,268 eligible patients, the incidence of CKD was 9.65% (5,722/59,268), and the overall mortality rate was 8.05% (4,770/59,268). The mortality in CKD patients was significantly higher than that in normal kidney function patients (12.48% vs. 7.57%, *P* < 0.001) (Supplementary Table 1).

### CD4/CD8 Ratio Recovery Was Association With Chronic Kidney Disease Cumulative Incidence

In our study, CD4/CD8 ratio recovery in only 11.72% (6,947 of 59,268) of patients when the restoration cutoff point was defined as 1.0 (defined as “CD4/CD8 ratio normalized” in many other studies). In addition, 28.98% (17,174 of 59,268) of patients with a CD4/CD8 ratio recovery when the cutoff point was defined as 0.7 (Supplementary Table 2). Univariate Cox regression analysis showed that patients whose CD4/CD8 ratio did not recover to 0.7 (HR = 2.87, 95% CI 2.67–3.08) had a higher risk of developing CKD than those did not recover to 1.0 (HR = 1.33, 95% CI 1.22–1.46) (Supplementary Table 2). Therefore, we defined 0.7 as the CD4/CD8 ratio recovery cutoff point in this study for further analyses.

**FIGURE 2:**
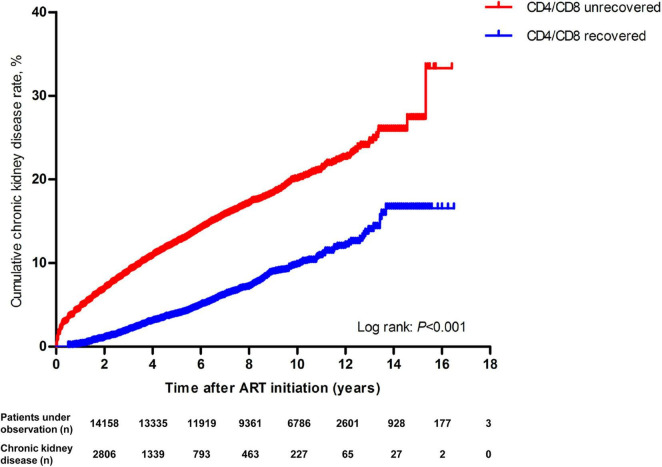
Kaplan–Meier analysis of cumulative incidence of chronic kidney disease for HIV patients receiving ART, grouped by CD4/CD8 ratio recovery (The statistical significance was measured by log-rank test).

Kaplan–Meier analysis showed that patients with an unrecovered CD4/CD8 ratio had a significantly higher cumulative CKD incidence than the recovery patients over the 17-year follow-up period (log-rank test: *P* < 0.001, Figure 2). Cox regression also showed that unrecovered CD4/CD8 ratio patients had a higher risk for CKD than recovery patients (aHR = 2.84, 95% CI 2.63–3.06, *P* < 0.001) (Supplementary Figure 1, red line).

We used 1:1 PSM analysis to match variables between the recovered and unrecovered CD4/CD8 ratio groups to minimize potential biases. A caliper of 0.00001 was set to ensure that all characteristic variables and ART regimen were properly matched between the two groups. Finally, 12,112 CD4/CD8 ratio recovery patients and 12,112 CD4/CD8 ratio unrecovered patients were included. Chi-squared test was subsequently performed to evaluate the effectiveness of the PSM. The results showed that the matched variables no longer exhibited statistically significant differences between the two groups after matching (data not shown). Cox regression analysis showed that unrecovered CD4/CD8 ratio patients had a higher risk for CKD than recovery patients (aHR = 3.13, 95% CI 2.85–3.45, *P* < 0.001) (Supplementary Figure 1, blue line). The results before and after PSM showed a strong relationship between the CD4/CD8 ratio and CKD.

### Relationship of Antiretroviral Therapy Regimen and CD4/CD8 Ratio Recovery or Chronic Kidney Disease Incidence

#### CD4/CD8 Ratio Recovery Among Different Antiretroviral Therapy Regimen Groups

In Supplementary Table 3, CD4/CD8 ratio recovery rate among the three ART regimen groups were significantly different, and the NNRTI-based group had the highest CD4/CD8 ratio recovery rate, followed by the PI-based and INSTI-based groups. While the CD8 also significantly decreased in the NNRTI-based group. However, the CD4 cell count showed highest increased in the PI-based group, followed by the NNRTI-based and INSTI-based groups. Moreover, median follow-up period and eGFR differences among the three ART regimen groups also showed statistically significance. Table 2 showed the changes in the CD4/CD8 ratio and the CD4 or CD8 cell count at CD4/CD8 recovery between the NNRTI-based and PI-based groups were significantly different (the CD4 cell count at the CD4/CD8 ratio recovered NNRTI-based group was lower than that in the PI-based group, while the CD8 cell count was higher).

**TABLE 2:** CD4/CD8 ratio recovery among different ART regimen groups [Medium (IQR)].

Variable	NNRTI-based	PI-based	INSTI-based	P*
Change in CD4/CD8 ratio	0.49 (0.36–0.63)	0.47 (0.34–0.61)	0.47 (0.36–0.61)	<0.001^#^
Time for CD4/CD8 ratio recovery, years	1.07 (0.49–2.89)	1.18 (0.49–3.00)	0.94 (0.30–1.97)	0.10
CD4 cell count at CD4/CD8 ratio recovery, cells/μL	502.24 (384.58–643.74)	550.13 (420.18–702.75)	552.50 (421.00–709.00)	<0.001^#^
CD8 cell count at CD4/CD8 ratio recovery, cells/μL	588.02 (441.47–766.23)	644.22 (480.77–841.77)	627.00 (515.50–831.00)	<0.001^#^

*P by non-parametric tests.

*#Pair wise comparison showed the difference was statistically significant only between the NNRTI-based and the PI-based group.*

#### Antiretroviral Therapy Regimens Was Association With CD4/CD8 Ratio Recovery

Cox regression analysis showed that the patients treated with NNRTI-based (aHR = 0.58, 95% CI 0.52–0.65, *P* < 0.001) or PI-based (aHR = 0.61, 95% CI 0.54–0.68, *P* < 0.001) regimens had a lower risk for unrecovered CD4/CD8 ratios than patients treated with INSTI-based regimens in all participants (Supplementary Figure 2, red line).

Because the number of patients varies greatly among the three ART regimen groups and some demographic characteristics were significantly different among the ART regimen groups (data not shown), we used 1:1:1 PSM analysis to match variables among these three groups to minimize potential biases. First, a caliper of 0.00001 was set to ensure that all demographic variables were properly matched between the NNRTI-based and PI-based groups, 13,159 NNRTI-based and 13,159 PI-based patients were included. Then, 13,159 NNRTI-based patients were properly matched to INSTI-based patients, 372 NNRTI-based and 372 INSTI-based patients were included at a caliper of 0.00001; similarly, 380 PI-based and 380 INSTI-based patients were also included at a caliper of 0.0000005. Finally, the three ART regimen groups of matched subjects were integrated, and a final number of 1,098 patients (366 NNRTI-based, 366 PI-based and 366 INSTI-based) were included. Chi-squared test was subsequently performed to evaluate the effectiveness of the PSM. The results showed that these demographic variables no longer exhibited statistically significant differences between each pair of ART regimen groups after matching (data not shown).

Cox regression analysis showed that, compared with the INSTI-based regimen, the NNRTI-based (aHR = 0.56, 95% CI 0.47–0.67, *P* < 0.001) or PI-based (aHR = 0.61, 95% CI 0.52–0.72, *P* < 0.001) regimen had a lower risk for unrecovered CD4/CD8 ratios after PSM (Supplementary Figure 2, blue line).

#### Antiretroviral Therapy Regimens Was Association With Chronic Kidney Disease Cumulative Incidence

**FIGURE 3:**
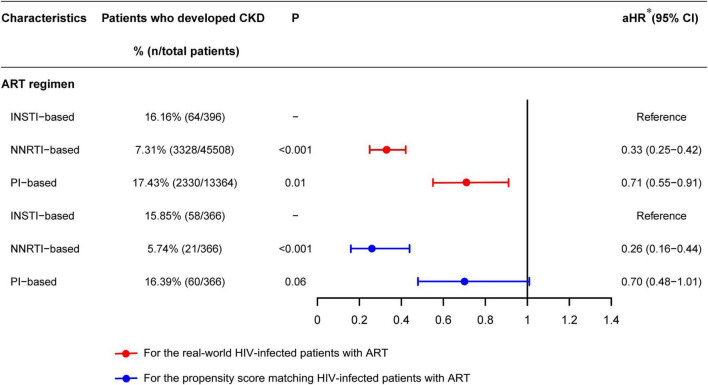
Forest plots of multivariable Cox regression analysis of the effect of ART regimen on chronic kidney disease among HIV patients receiving ART. [*aHR, adjusted hazard ratio, adjusted by ART regimen, CD4/CD8 ratio recovery, sex, marital status, age at HIV diagnosis, age at ART initiation, HIV transmission route, WHO HIV disease stage, baseline BMI, baseline CD4 cell count, baseline CD8 cell count, baseline CD4/CD8 ratio, HBV infection, HCV infection, TB infection in the past year, and other opportunistic infections in the past 3 months (including thrush, hairy leukoplakia, esophageal candidiasis, PCP, toxoplasmic encephalitis, Cytomegalovirus infection, disseminated mycosis and extrapulmonary TB)].

As shown in Figure 3, after adjustment for a collection of predefined and forward-selection variables, Cox regression analysis indicated that the NNRTI-based (aHR = 0.33, 95% CI 0.25–0.42, *P* < 0.001) or PI-based (aHR = 0.71, 95% CI 0.55–0.91, *P* = 0.01) regimen had a lower incidence of CKD than that of INSTI-based regimen (Figure 3, red line). The PSM analysis also showed that the NNRTI-based (aHR = 0.26, 95% CI 0.16–0.44, *P* < 0.001) ART regimen had a lower incidence of CKD than that of INSTI-based regimen (Figure 3, blue line).

#### Stratified Analysis of Antiretroviral Therapy Regimens

**FIGURE 4:**
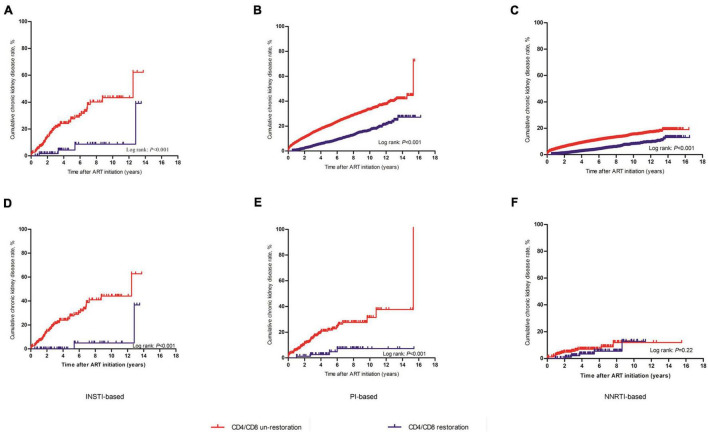
Kaplan-Meier analysis of cumulative incidence of chronic kidney disease for HIV patients receiving ART, grouped by ART regimen. **(A)** The whole research patients treated with INSTI-based regimen. **(B)** The whole research patients treated with PI-based regimen. **(C)** The whole research patients treated with NNRTI-based regimen. **(D)** The PSM patients treated with INSTI-based regimen. **(E)** The PSM patients treated with PI-based regimen. **(F)** The PSM patients treated with NNRTI-based regimen. The statistical significance was measured by log-rank test.

Stratified analysis of different ART regimens was used to evaluate the relationship of CD4/CD8 ratio recovery and CKD in three subgroups of ART regimens using the Kaplan–Meier method. In all three ART regimen subgroups, the cumulative incidences of CKD in the CD4/CD8 ratio unrecovered group were significantly higher than those in the recovered group (Figures 4A–C). The largest difference in CKD incidence between the CD4/CD8 ratio recovered and unrecovered groups was found in the INSTI-based subgroup (Figure 4A). Additionally, the highest CKD incidence rate (7/100 person-years, 95% CI 5–8) was found among patients treated with the INSTI-based regimen and the CD4/CD8 ratio was unrecovered, and was significantly higher than the CD4/CD8 ratio recovery patients (Supplementary Table 4). Moreover, the stratified analysis after PSM also showed that in PI-based and INSTI-based ART regimen subgroups, the cumulative incidences of CKD in the CD4/CD8 ratio unrecovered group were significantly higher than those in the recovery group (Figures 4D–F). However, no significant difference was seen in the NNRTI-based group (log-rank test: *P* = 0.22, Figure 4F).

## Discussion

This is a real-world study with a 17-year observation period and a large sample size that was conducted on HIV-positive individuals in Guangxi, China to explore the relationship of the CD4/CD8 ratio recovery and CKD. The study indicated that the incidence of CKD was 9.65% in HIV patients with ART, which is lower than those (15.4–16.8%) in several previous studies (Cheung et al., 2007; Wyatt et al., 2007; Yanagisawa et al., 2011; Cao et al., 2013). The main reason for this difference is that the rates in previous studies were the overall incidence of CKD, whereas our study excluded HIV patients with an abnormal baseline eGFR, which leads to the relatively lower incidence of CKD. This is the first study to estimate the CKD incidence of 9.65% in Guangxi over a median ART period of 4 person-years. The incidence estimate is important, as it emphasizes a substantial burden of CKD among these patients. Our study also showed that patients with CKD had a higher risk of death, which is consistent with the results of previous research, indicating that kidney disease can significantly increase the risk of death in HIV-infected individuals (Sarfo et al., 2013; Mallipattu et al., 2014).

CD4 cell count has been considered as an influencing factor for NCDs in HIV-infected patients. With ART, the CD4 cell count in most patients gradually normalizes, but the CD8 cell count might remain at a high level, which leads to the CD4/CD8 ratio remaining low and difficult to recover to normal levels. In our study, the CD4 cell count did not necessarily raise to 500 cells/μL or above in patients whose CD4/CD8 ratio recovered to 0.7 (Table 2). Previous studies have showed that a low CD4/CD8 ratio in HIV-infected patients identifies greater immune defects, which might be at a higher risk of mortality, various infections, including kidney infection (Trickey et al., 2017). In recent years, growing evidence suggests that the CD4/CD8 ratio should be considered as an emerging biomarker for HIV-related diseases. Our study revealed that, among HIV patients with normal baseline kidney function, CD4/CD8 ratio recovery was strongly associated with the incidence of CKD, which was supported by the overall patient-wide Cox regression analysis and PSM analysis designed to reduce biases in the distribution of potentially confounding variables. Furthermore, our study found that patients whose CD4/CD8 ratio failed to recover to 0.7 may have poor immune function, leading to a higher risk of CKD. These findings have significant implications for the relationship between CD4/CD8 ratio and NCDs in HIV patients, and are an important complement to previous studies (Castilho et al., 2019; Gojak et al., 2019). In addition, we found that the median CD4 cell count in the NNRTI-based group was lower than that in PI-based or INSTI-based groups when CD4/CD8 ratio recovered, however, the incidence of CKD in NNRTI-based group was the lowest (Supplementary Table 3), indicating that although CD4 cell count of some participants did not return to normal level, the risk of CKD could be partially reduced as long as the CD4/CD8 ratio was restored. That is, the CD4/CD8 ratio may be a more important reflection for the CKD incidence of HIV-1 infected patients than the CD4 cell count alone.

Previous studies have reported that INSTI-based ART was associated with a greater increase in the CD4/CD8 ratio (Masia et al., 2016; Serrano-Villar et al., 2020). In the present study, we further explored whether there was a significant difference in the ART regimens between the recovered CD4/CD8 group and the non-recovered group. We found that the INSTI-based regimen had the lowest CD4/CD8 ratio recovery rate at a cutoff of 0.7, which is inconsistent with two previous studies, indicating the INSTI-based regimen had higher CD4/CD8 ratio normalization rates than NNRTI-based or PI-based regimens (Herrera et al., 2020; Serrano-Villar et al., 2020). Moreover, previous studies have showed the largest differences in CD4/CD8 ratio trajectories were driven by changes in the CD8 cell counts (Serrano-Villar et al., 2017, 2020). They found the adjusted mean CD8 cell counts was highest in PI-based regimen, and a higher rate of CD4/CD8 ratio normalization with efavirenz, which was driven by greater CD8 cell decline (Serrano-Villar et al., 2017, 2020). However, our study found that the median CD8 decreased only in the NNRTI-based regimen, and this regimen had the highest CD4/CD8 ratio recovery rate. The PI-based and INSTI-based regimens had relatively lower CD4/CD8 ratio recovery rate, and the median CD8 were almost unchanged. Different CD4/CD8 ratio cutoff values as well as different ART policies or treatment details in different countries may be the reasons for these inconsistent results.

Antiretroviral drugs have been shown to be associated with NCDs such as diabetes and obesity among HIV patients (Coetzee et al., 2019). Several studies have also confirmed an association of CKD incidence with PIs (Yombi et al., 2014; Mocroft et al., 2016). Our study showed that the association between CD4/CD8 ratio recovery and reduced CKD incidence was consistently observed in all three ART regimen groups. It is worth noting that the benefit of CD4/CD8 ratio recovery on CKD incidence was most significant in the INSTI-based group, this result was also confirmed by PSM analysis. This provides further support for the finding because the patients with the INSTI-based regimen in our study had the lowest CD4/CD8 ratio recovery rate. If CD4/CD8 ratio recovery is related with CKD incidence, patients with the least desirable CD4/CD8 recovery need to be focused. In particular, the CKD incidence in PI-based or INSTI-based regimens was significantly different between the CD4/CD8 ratio recovered and unrecovered groups, while there was no significant difference in NNRTI-based regimen. This suggests that patients using PI-based or INSTI-based regimens should pay more attention to CD4/CD8 ratio recovery to minimize the risk of CKD. In addition, PIs have been widely recognized as nephrotoxic, but there are few studies on whether INSTIs have side effects on the kidney (Yombi et al., 2014; Mocroft et al., 2016). In this sense, our study provides new clues that PI and INSTI may be nephrotoxic.

It should be noted that our study has some limitations. First, this was a retrospective cohort study, and we could not control some confounding factors, such as the use of medications other than the ART regimens, which might be associated with renal toxicity. Second, due to the lack of viral suppression data from the participants, it was not possible to determine whether patients’ viral load was suppressed when the CD4/CD8 ratio recovered to 0.7. Despite these limitations, to the best of our knowledge, this is the first large cohort study in a real-world setting to investigate the relationship of CD4/CD8 ratio recovery and CKD incidence in HIV-infected patients receiving ART. The recovery of the CD4/CD8 ratio is better, and the incidence of CKD is lower in HIV patients treated with NNRTI-based regimens than in those treated with PI-based or INSTI-based regimens. These results highlight the importance of monitoring the CD4/CD8 ratio in patients receiving ART, especially with PI-based or INSTI-based regimens.

## Data Availability Statement

The original contributions presented in the study are included in the article/Supplementary Material, further inquiries can be directed to the corresponding author/s.

## Ethics Statement

The studies involving human participants were reviewed and approved by the Human Research Ethics Committee of Guangxi Medical University. Written informed consent for participation was not required for this study in accordance with the National Legislation and the Institutional Requirements.

## Author Contributions

JJ, LY, SQ, and HaL conceived and designed the study. FQ, JJ, QL, and WH conducted the data analysis and literature review and drafted the manuscript. FQ, QL, WH, DW, KH, and KL involved in the study supervision, data collection, and interpretation of the data. FQ, RC, JL, BL, and HuL assisted with data management and data analysis. All authors contributed to the revision of the manuscript and approved the final version.

## Conflict of Interest

The authors declare that the research was conducted in the absence of any commercial or financial relationships that could be construed as a potential conflict of interest.

## Publisher’s Note

All claims expressed in this article are solely those of the authors and do not necessarily represent those of their affiliated organizations, or those of the publisher, the editors and the reviewers. Any product that may be evaluated in this article, or claim that may be made by its manufacturer, is not guaranteed or endorsed by the publisher.
