# Supplementary material for: Association Between CD4/CD8 Ratio Recovery and Chronic Kidney Disease Among Human Immunodeficiency Virus-Infected Patients Receiving Antiretroviral Therapy: A 17-Year Observational Cohort Study
Source: Front Microbiol. 2022 Feb 10;13:827689. doi: 10.3389/fmicb.2022.827689 (PMC8867036; doi:10.3389/fmicb.2022.827689)
Supplement: Supplementary file 1 [file Data_Sheet_1.pdf]

Supplementary Material

1 Supplementary Tables

Supplementary Table 1. Relationship between kidney function and mortality in HIV/AIDS patients receiving ART

Survival situation	Chronic kidney disease n (%)	Normal kidney function n (%)	Total n (%)	χ^2	*P*
Death	714 (12.48)	4056 (7.57)	4770 (8.05)	167.95	<0.001
Survival	5008 (87.52)	49490 (92.43)	54498 (91.95)		
Total n (%)	5722 (9.65)	53546 (90.35)	59268 (100.00)		

Supplementary Table 2. Effect of the CD4/CD8 ratio recovery at different cutoff point on chronic kidney disease among HIV patients receiving ART.

CD4/CD8 ratio recovery cutoff point	Total patients n (%)	Patients who developed chronic kidney disease n (%)	HR* (95%CI)	*P*
CD4/CD8 ratio recovery at a cutoff of 1.0				<0.001
Yes	6947 (11.72)	574 (8.26)	1.00	-
No	52321 (88.28)	5148 (9.84)	1.33 (1.22-1.46)	<0.001
CD4/CD8 ratio recovery at a cutoff of 0.7				<0.001
Yes	17174 (28.98)	922 (5.37)	1.00	-
No	42094 (71.02)	4800 (11.40)	2.87 (2.67-3.08)	<0.001

*HR: hazard ratio.

Supplementary Table 3. Clinical characteristics of study population [Medium(IQR)]

Variable	Total patients	NNRTI-based	PI-based	INSTI-based	*P*
CD4/CD8 ratio recovery rate, n (%)	17174 (28.98)	13497 (29.66)	3613 (27.04)	64 (16.16)	<0.001

CD8 cell count changes, cells/μL	-61 (-372-244)	-80 (-394-216)	6 (-294.8-328.8)	12 (-411.3-375.8)	<0.001*
CD4 cell count changes, cells/μL	192 (85-324)	188 (84-318)	208 (90.25-348)	145 (59-287.5)	<0.001*
Follow-up period, years	4.42 (2.17-7.08)	4.33 (2.09-7.00)	4.58 (2.25-7.30)	1.87 (0.92-4.17)	<0.001*
eGFR, mL/min per 1.73 m^2	119.22 (104.25-140.64)	118.60 (104.14-139.01)	121.59 (104.75-146.77)	118.06 (103.90-138.13)	<0.001*

**P* by non-parametric tests

Supplementary Table 4. The chronic kidney disease rate of HIV patients receiving ART, group by ART regimen

ART regimen	CD4/CD8 ratio recovery	Total patients n	Chronic kidney disease n (%)	Person-years	Chronic kidney disease /100 Person-years (95%CI)	*P**
INSTI-based	Yes	64	4 (6.25)	296.97	1.35 (0.06-2.63)	<0.001
	No	332	60 (18.07)	920.48	6.52 (4.91-8.13)	
PI-based	Yes	3613	365 (10.10)	22727.31	1.61 (1.45-1.77)	<0.001
	No	9751	1965 (20.15)	43700.78	4.50 (4.30-4.69)	
NNRTI-based	Yes	13497	553 (4.10)	81519.69	0.68 (0.62-0.73)	<0.001
	No	32011	2775 (8.67)	135978.31	2.04 (1.97-2.12)	

**P* by log-rank test.

2 Supplementary Figures

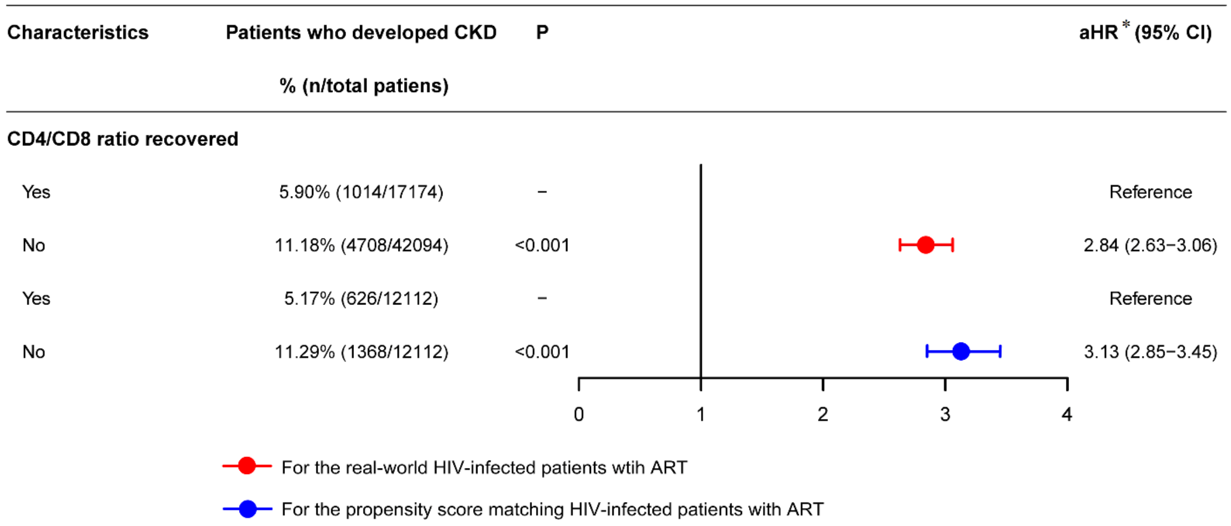

Supplementary Figure 1. Forest plots of multivariable Cox regression analysis of the effect of CD4/CD8 ratio recovery on chronic kidney disease among HIV patients receiving ART. (*aHR: adjusted hazard ratio, adjusted by CD4/CD8 ratio recovery, ART regimen, sex, marital status, age at HIV diagnosis, age at ART initiation, HIV transmission route, WHO HIV disease stage, baseline BMI, baseline CD4 cell count, baseline CD8 cell count, baseline CD4/CD8 ratio, Hepatitis B Virus (HBV) infection, Hepatitis C Virus (HCV) infection, TB infection in the past year, and other opportunistic infections in the past three months (including thrush, hairy leukoplakia, esophageal candidiasis, Pneumocystis carinii pneumonia (PCP), toxoplasmic encephalitis, Cytomegalovirus infection, disseminated mycosis and extrapulmonary TB)).

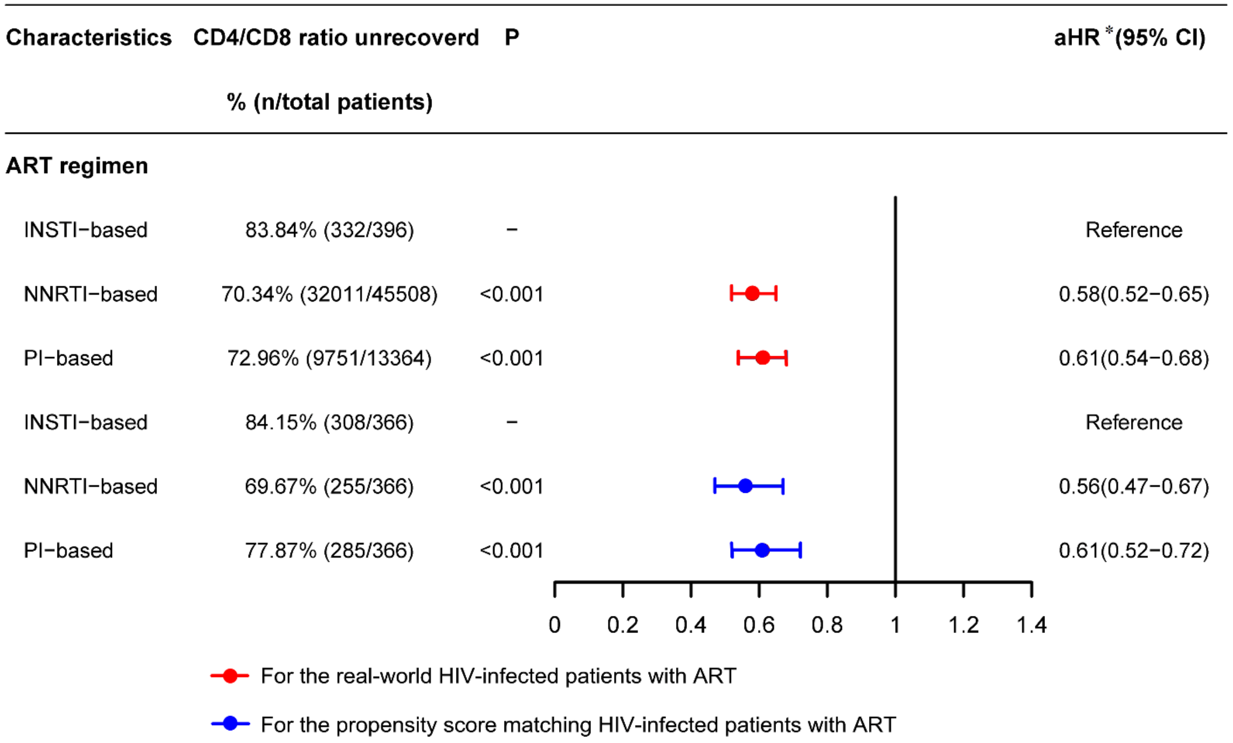

Supplementary Figure 2. Forest plots of multivariable Cox regression analysis of the effect of ART regimen on CD4/CD8ratio recovery among HIV patients receiving ART.(*aHR: adjusted hazard ratio, adjusted by ART regimen, sex, marital status, age at HIV diagnosis, age at ART initiation, HIV transmission route, WHO HIV disease stage, baseline BMI, baseline CD4 cell count, baseline CD8 cell count, baseline CD4/CD8 ratio, HBV infection, HCV infection, TB infection in the past year, and other opportunistic infections in the past three months (including thrush, hairy leukoplakia, esophageal candidiasis, PCP, toxoplasmic encephalitis, Cytomegalovirus infection, disseminated mycosis and extrapulmonary TB)).
